# CPG-Based Gait Generation of the Curved-Leg Hexapod Robot with Smooth Gait Transition

**DOI:** 10.3390/s19173705

**Published:** 2019-08-26

**Authors:** Long Bai, Hao Hu, Xiaohong Chen, Yuanxi Sun, Chaoyang Ma, Yuanhong Zhong

**Affiliations:** 1 State Key Laboratory of Mechanical Transmission, Chongqing University, Chongqing 400044, China; 2 College of Mechanical Engineering, Chongqing University, Chongqing 400044, China; 3 School of Microelectronics and Communication of Engineering, Chongqing 400044, China

**Keywords:** legged locomotion, legged robot, hexapod robot, gait planning, bionic locomotion control, CPG

## Abstract

This paper presents a novel CPG-based gait generation of the curved-leg hexapod robot that can enable smooth gait transitions between multi-mode gaits. First, the locomotion of the curved leg and instability during the gait transitions are analyzed. Then, a modified Hopf oscillator is applied in the CPG control, which can realize multiple gaits by adjusting a simple parameter. In addition, a smooth gait switching method is also proposed via smooth gait transition functions and gait planning. Tripod gait, quadruped gait, and wave gait are planned for the hexapod robot to achieve quick and stable gait transitions smoothly and continuously. MATLAB and ADAMS simulations and corresponding practical experiments are conducted. The results show that the proposed method can achieve smooth and continuous mutual gait transitions, which proves the effectiveness of the proposed CPG-based hexapod robot control.

## 1. Introduction

Compared with wheeled robots, legged robots have a stronger ability to adapt to different kinds of rough terrain and obstacles. Therefore, they have attracted much attention from scientists and engineers since the 1990s [1,2,3,4]. The hexapod robot, which is inspired by hexapod animals, is one of the most representative legged robots that can obtain satisfactory stability and flexibility in a complex environment [5,6,7]. Most of the hexapod robots are inspired by the stick insects which have six limbs consisting of coxa, femur and tibia, and each limb has three rotating joints connecting the body and the coxa, the coxa and the femur, and the femur and tibia [8]. In different situations, the hexapod robots, like the stick insects, have different numbers of legs supporting the body and different stride sequences, called gaits [9]. Three different gaits are usually identified in insects: the tripod gait, the quadruped gait and the wave gait. Different from these hexapod robots, the RHex robots [10,11], developed by a multidisciplinary and multi-university U.S. government DARPA-funded effort, replace the multi-jointed limbs with half-circle shaped compliant legs. Compared with the robots with multi-jointed limbs, the hexapod robot with curved legs increases its mobility while maintaining higher adaptability than wheeled robots. There is only one rotating joint to connect each curved leg with the body, which makes it easier to control the locomotion. The studies of Garcia also suggest that it is more efficient for the robot to propel by rolling contact than by tip contact [12]. RHex robots can achieve high speed running based on tripod gait in terrain and stair climbing [13]. Besides, the researchers also realized the leaping and flipping of RHex via the Spring-Loaded Inverted Pendulum (SLIP) [14,15] dynamic model.

Based on the original RHex-type robots, Haynes et al. developed the X-RHex [16,17] with a more compact structure and higher energy density through the analysis of structural reliability and energy efficiency. Lin et al. presented an Rolling Spring-Loaded Inverted Pendulum (R-SLIP) dynamics model that takes the rolling contact into account, which can realize the leaping of an RHex-style hexapod robot [18,19]. By imitating the gait of cockroaches, an obstacle-crossing gait has been proposed by Chou, which can enable the obstacle-crossing height of the RHex to reach twice the diameter of its curved leg [20]. To further enhance the robotic performance, Zhang et al. have developed an RHex-style amphibious robot with deformable curved legs, i.e., the AmphiHex-I [21]. The curved leg of which is made up of rod pieces connected by ropes. When walking on the land, the rope is tightened to make the leg stay curved; when propelling under the water, the rope is loosened to make the leg become a flexible paddle.

Although a great number of studies have verified the suitability of hexapod robots in various complex environments, the multiple degrees of freedom (DOF) coordinated gait control, as well as the smooth transition between multi-mode gaits, remain open topics for researchers. The animal biology and neurophysiology studies reveal that the rhythmic limb activities of animals are governed by the central pattern generator (CPG) located in the spinal cord without sensory feedback or regulation command from the brain-stem level [22]. Compared with the conventional methods such as behavior-based control method and model-based control method, CPG-based robotic controls do not require accurate and sensitive feedbacks and can achieve multi-mode gaits via a single network model, which greatly reduces the complexity of multi-mode robotic control. Animal CPG can be mathematically modeled by a group of neural oscillators formulated by coupled nonlinear differential equations, and the specific frequency, amplitude, and phase relationship of the output quasi-periodic waveforms can be precisely tuned by adjusting the parameters of CPG networks [23].

To imitate the control of CPG on animal rhythmic locomotion, plenty of CPG models have been built for different types of robots [24,25]. The first practical CPG model was proposed by Matsuoka in 1987 [26], which could enable the steady walking of a Tekken II quadruped robot [27]. Afterwards, the CPG-based locomotion control has been successfully applied to a variety of bioinspired robots such as the snake-like robot [28,29], the fish-like robot [30,31], the biped robot [32,33], the quadruped robot [34,35] and the hexapod robot [36,37]. Minati et al used FPAA-based oscillators set up a hierarchical CPG network for an ant-like hexapod robot. Six oscillators form a central pattern generator to produce the global leg coordination pattern, and each node is coupled with a local pattern generator devoted to generating the trajectory of one leg. Several kinds of identified gaits and continuous generalized gait are yielded through changing the topology and strengths of the connections coupling the oscillators [38,39,40]. This method has achieved good results in hexapod robots with multi-jointed limbs.

Although replacing the multi-jointed limbs with the curved leg promotes the mobility and simplifies the control, the freedom of each leg is reduced and the trajectory is not adjustable. It is hard for hexapod robots with curved-legs to continuously maintain their stability through the coordination of the leg joints. Different from ant-like robots, the joints of the hexapod robots with curved-legs rotate in one direction instead of swinging back and forth, so a relatively complex mapping relationship from the output sine-like wave of CPG to the joint space is needed, and distortion of the waveform will have a greater impact on the smoothness of the joint’s rotational trajectory. In summary, there are three areas that need to be addressed when applying the CPG to the gait generation and gait transitions of hexapod robots with curved-legs:

(1) Most of the CPG models could only generate symmetric waveforms, which requires a complicated mapping between the CPG model and various gaits within asymmetric phases.
(2) Distortion of waveform, for example, the wave will have a sharp point and oscillate multiple times on the same side of the X-axis, should be avoided so as not to cause serious impact on the smoothness of motor rotation.
(3) During the gait transitions, the simplified structure of the curved-leg make it difficult to keep the supporting stability by adjusting the robotic posture. Compared with multi-jointed limbs, the hexapod robots with curved-legs need to void the unstable supporting situation based on suitable gait planning for these robots.

Therefore, in this paper, a modified Hopf-based CPG model is proposed, which can generate the required control signal waveforms corresponding to the tripod gait, the quadruped gait, and the wave gait. In addition, aimed at keeping the robotic supporting stability and avoiding the impact on the motors, a stable and smooth gait transition method is put forward by stability analysis and transition planning between these gaits. Finally, Adams simulations and practical experiments are conducted to investigate fully and verified the performance of the proposed method.

The rest of this paper is organized as follows: Section 2 introduces the analysis of the locomotion and the supporting stability of our hexapod robot; Section 3 presents the design of the proposed CPG network as well as the realization of smooth gait transitions; Section 4 shows the simulation and experiments and Section 5 concludes the paper.

## 2. Locomotion and Stability Analysis

**Figure 1:**
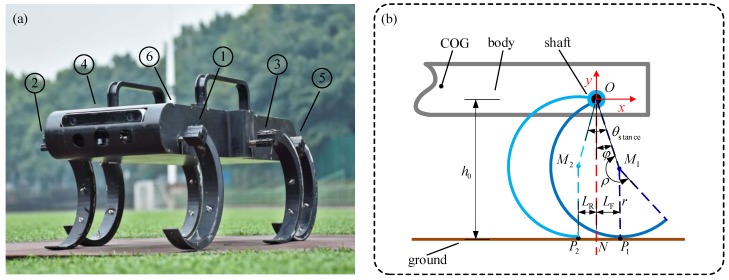
The hexapod robot with curved legs: (**a**) The prototype consists of a body and six curved-legs, the six legs are numbered 1 to 6 in order from left to right and from front to rear, each leg is connected to the body by a rotating joint.; (**b**) Locomotion analysis of one curved leg: *r* is the radius, *ρ* is the central angle, he curved leg lands at *P*_1_ and departs the ground at *P*_2_ after a rolling contact, *L*_F_ is the front bias; and *L*_R_ is the rear bias, *ϕ* is the bias angle and its initial value is *ϕ*_0_.

In Figure 1a, each curved leg of the hexapod robot is actuated via an electric motor, and are sequentially numbered Leg 1, Leg 2, Leg 3, Leg 4, Leg 5, Leg 6. Figure 1b shows the diagrammatic drawing of the curved leg, where r represents the radius and ρ represents the central angle. To avoid interference between different legs, the widths of the front and the rear part of the robot are relatively narrower than that of the middle part, where the width of the front/rear part is $W_{n}$, and the middle width is $W_{b}$. The longitudinal distance between each leg is D. Table 1 lists the detailed structure parameters.

### 2.1. Locomotion Analysis

The locomotion of one single leg can be divided into two phases, the stance phase, and the swing phase. The stance phase enables the robot to propel while the swing phase makes the leg recirculate. During one rotation cycle, the stance and the swing angles are $\theta_{stance}$ and $\theta_{swing}$, respectively.

Figure 1b shows the start and end positions of the curved leg in the stance phase. The curved leg lands at $P_{1}$ and departs the ground at $P_{2}$ after a rolling contact. Let $L_{F}$ represents the distance between $P_{1}$ and the joint shift in the horizontal direction, i.e., the front bias; and $L_{R}$ represents the distance between $P_{2}$ and the joint shift in the horizontal direction, i.e., the rear bias; and φ is the angle between the vertical direction and the line that connects the joint shift and the center of the curved leg, namely, the bias angle. The initial value is of φ is $\varphi_{0}$.

From the geometric relationship of the robot, it can be known that $\varphi_{0}$ determines the parameter $\theta_{stance}$, thereby determining the propelling distance L during the stance phase. Let the speed of robot in the horizontal and the vertical direction be $v_{X}$ and $v_{Y}$, respectively; the average advance speed is of the robot in one walking cycle is V; and the height of the robot during landing is $h_{0}$.

Because $P_{1}$ and $P_{2}$ are symmetrical along the vertical line passing through the point O, it can be found that:

(1) $$\theta_{stance}=2\varphi_{0}$$

During the stance phase, the bias angle varies from $\varphi_{0}$ to $-\varphi_{0}$, at any time. Therefore, the displacement and the velocity of the joint point O in the horizontal and numerical vertical directions can be expressed by:

(2) $$x=r(\varphi_{0}-\varphi +\sin\varphi_{0}-\sin\varphi )$$

(3) y=r(1+cosφ)

(4) $$v_{X}=\frac{dx}{dt}=-r\dot{\varphi }(1+\cos\varphi )$$

(5) $$v_{Y}=\frac{dy}{dt}=-r\dot{\varphi }\sin\varphi$$

From the symmetrical relationship, it can be found that $L_{F}$ equals $L_{R}$, the propulsion distance L of the curved leg is the sum of $L_{F}$, $L_{R}$ and the rolling distance:

(6) $$L_{F}=L_{R}=r\sin\varphi_{0}$$

(7) $$L=2r(\varphi_{0}+\sin\varphi_{0})$$

When the robot propels, the contact between the legs and the ground is discontinuous. The ratio of the stance phase to the full rotation cycle is defined as the duty factor ε. Therefore, for common gaits: tripod gait is with the duty factor of 0.5, quadruped gait is with the duty factor of 2/3 and wave gait is with the duty factor of 5/6. Assume that the quantity of legs in one stance phase is n, then the relationship between n and duty factor can be expressed by:

(8) n=6ε

Assume that the time the gait cycle once is T (sec), cosisting of the stance stage and the swing stage, and the average rotational speed of one joint is N (rpm). In one cycle, one leg goes through a stance phase and the forward distance is L, and during the swing phase of this leg, the forward distance is:

(9) $$L_{swing}=\frac{1-\varepsilon }{\varepsilon }L$$

Then the full forward distance is:

(10) $$L_{full}=L+L_{swing}=\frac{L}{\varepsilon }$$

The average advance speed V (m/s) of the robot can be obtained according to the relationship of cycle time and the average rotational speed:

(11) $$N=\frac{60}{T}$$

(12) $$V=\frac{L_{full}}{T}=\frac{NL_{full}}{60}=\frac{NL}{60\varepsilon }=\frac{Nr}{30\varepsilon }(\varphi_{0}+\sin\varphi_{0})$$

From Equations (8) and (12), it can be found that bigger n and less V can be achieved with a bigger duty factor ε.

### 2.2. Supporting stability of the gaits

**Figure 2:**
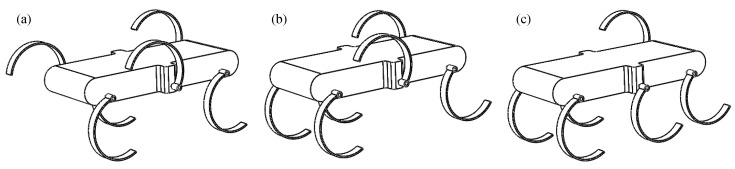
The supporting situations of the three kinds of gaits at a certain moment: (**a**) tripod gait: three legs in stance phase and three legs in swing phase at any time; (**b**) quadruped gait: four legs in stance phase and two legs in swing phase at any time; (**c**) wave gait: five legs in stance phase and one leg in swing phase.

Inspired by the various gaits of hexapods, this paper plans tripod, quadruped and wave gaits to deal with terrain of different complexity. Figure 2 shows the supporting situations of the three kinds of gaits at a certain moment, respectively. As shown in Figure 2a, when walking in the tripod gait, the six legs are divided into two groups to support and swing in turns, there are three legs in stance phase at any time; as shown in Figure 2b, when walking in the quadruped gait, the six legs are divided into three groups to support and swing in turns, there are four legs in stance phase at any time; as shown in Figure 2c, when walking in the wave gait, the six legs support and swing in turns, at any time, there are five legs in stance phase.

**Figure 3:**
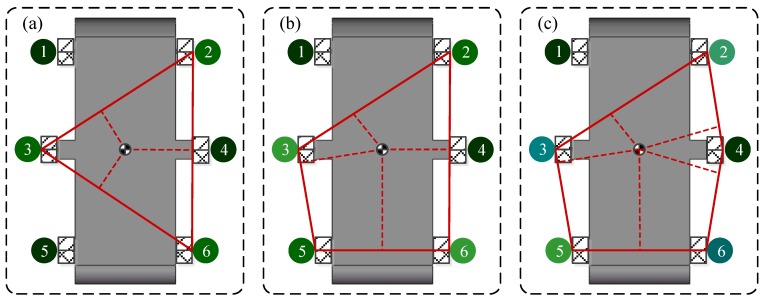
The supporting polygons: (**a**) tripod gait: the stable supporting area is the triangle formed by three supporting points; (**b**) quadruped gait: the stable supporting area is the quadrangle formed by four supporting points; (**c**) wave gait: the stable supporting area is the pentagon formed by five supporting points.

In this paper, the supporting stability is analyzed based on the Static Stability Margin (SSM) proposed by McGhee et al. [41,42]. According to this method, the contact point between the robot’s leg and the ground constitutes a stable support area. If the projection of the robotic centroid falls within this area, the robot is considered to be in a stable support state. The larger the distance between the projection point and the support boundary, the more stable it is considered. Figure 3 shows the supporting polygons formed by the supporting points at this moment. $d_{\mathrm{ij}}$ represents the distance from centroid projection to the supporting edge connecting the supporting points of Leg i and Leg j. These distances can be obtained from the structure parameters. Table 2 lists the distances of the tripod gait. Table 3 lists the distances of the quadruped gait and the wave gait.

According to Figure 3 and Table 2 and Table 3, it can be found that there are most supporting points and biggest supporting polygon in the wave gait, the supporting points of the tripod gait are the fewest and the supporting polygon of the tripod gait is the smallest. At the same speed input, the wave gait speed is the slowest, but the static support stability is the best, so it is generally used when the road surface conditions are extremely poor and the speed requirement is not high. Conversely, the tripod gait is the fastest, but the static support is the least stable, so it can be applied to situations where the speed needs to be high. The four-foot gait falls between these two gaits and can be applied to situations where the road surface is poor but speed is required.

### 2.3. Instability Analysis During Gait Transitions

**Figure 4:**
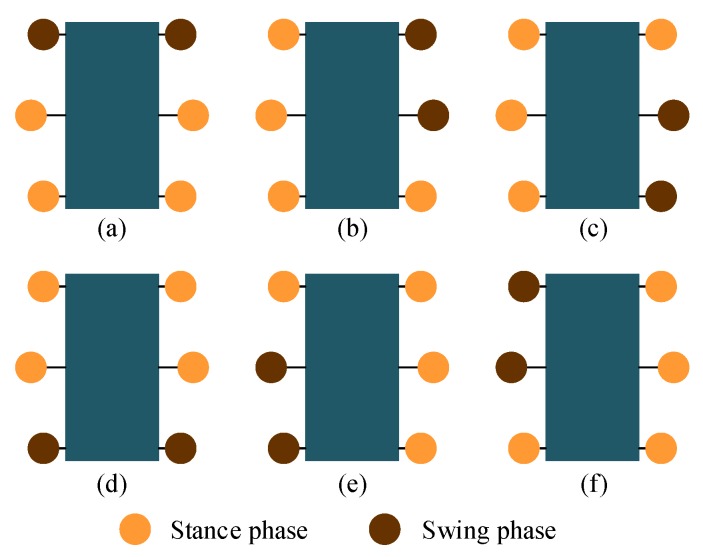
The six kinds of unstable states: (**a**) when leg 1 and leg 2 are in the swing phase at the same time, the body will fall forward; (**b**) when leg 2 and leg 4 are in the swing phase at the same time, the body will fall to the right front; (**c**) when leg 4 and leg 6 are in the swing phase at the same time, the body will fall to the right rear; (**d**) when leg 6 and leg 5 are in the swing phase at the same time, the body will fall backwards; (**e**) when leg 5 and leg 3 are in the swing phase at the same time, the body will fall to the left rear; (**f**) when leg 3 and leg 1 are in the swing phase at the same time, the body will fall to the left front.

During the gait transition, although the current output waveforms of the CPG will converge to the target stable waveforms smoothly and eventually, an unsuitable gait planning may put the robot in unstable states without enough supporting points because only one rotating joint in each leg makes it difficult to keep the projection of the robotic centroid falling within the stable support area through adjusting the posture. Based on the SSM method, the unstable supporting states of hexapod robots with curved-legs can be identified to be that the body would tilt to the corner where two adjacent legs are in swing phase at the same time (such as Leg 1 with Leg 2, Leg 2 with Leg 4, Leg 4 with Leg 6, Leg 6 with Leg 5, Leg 5 with Leg 3, Leg 3 with Leg 1), as shown in Figure 4. Taking Figure 4a as an example, if Leg 1 and Leg 2 are in the swing phase at the same time during the transitions, tilt forward.

To verify the effectiveness of the proposed gait planning, a dimensionless parameter χ is selected to characterize the degree of disorder during the transition. A series of points in a cycle on the time axis (0 to 1, the accuracy of 0.01) are selected as the starting point of the gait switching, and the degree of disorder of the gait switching from the point can be obtained by simulation. Its initial value is 1, once an unstable state mentioned previously happens during the transition, χ will increase 0.01. If the degree of disorder obtained by switching at any point in a cycle is 1, then this gait planning is suitable. If the degree of disorder obtained by switching at some point is greater than 1, then this gait planning is not suitable because it is not possible to ensure the supporting stability of the robot switching gait at any time.

## 3. CPG Network Model

### 3.1. Instability Analysis During Gait Transitions

A Hopf oscillator is selected to be the neuron oscillator of CPG networks in this paper owing to its following prominent features: fast rate of convergence, prominent robustness for disturbances, independently adjustable frequency and amplitude for its parameters have definite physical meaning.

**Figure 5:**
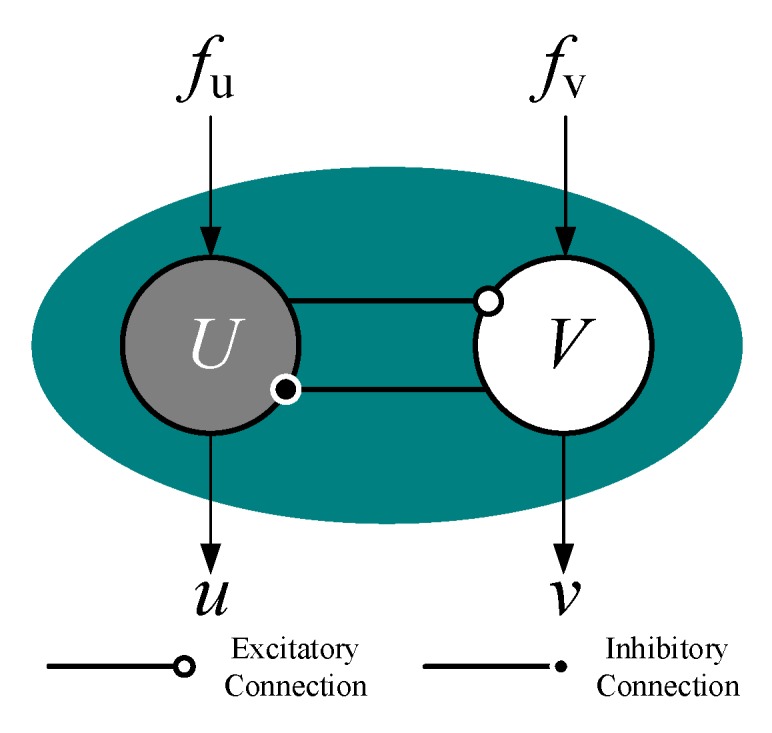
Hopf oscillator.

As shown in Figure 5, every oscillator consists of an excitatory neuron and an inhibitory neuron. The Hopf oscillator model can be defined as the following nonlinear differential equations:

(13) $$\dot{X}=\left[\begin{array}{c} \dot{u}\\ \dot{v} \end{array}\right]=\left[\begin{array}{c} \sigma (R^{2}-u^{2}-v^{2})u-2\pi \omega v+f_{u}\\ \sigma (R^{2}-u^{2}-v^{2})v+2\pi \omega u+f_{v} \end{array}\right]$$

where $X={\left[\begin{array}{cc} u & v \end{array}\right]}^{T}$ represents the state vector of the state equation; σ donates a positive constant of the speed of convergence; ω represents the frequency of the oscillator, which is determined according to the target rotation speed because they are proportional to each other; and R is the amplitude of the steady state oscillation, which is set as 1 to simply the mapping relationship; $f_{u}$ and $f_{v}$ represent the coupling term from other oscillators in the CPG networks, which will be of zero value when there is only one oscillator working.

**Figure 6:**
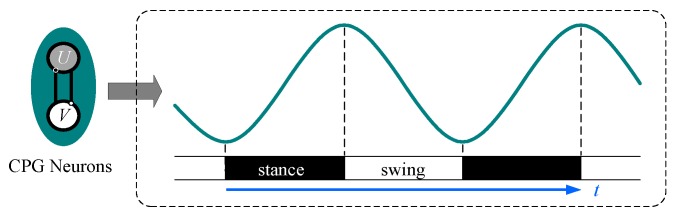
Correspondence between the oscillator output and robotic locomotion phase, he ascent stage of the signal is set corresponding to the stance phase, the declining stage is set corresponding to the swing phase.

The waveforms of state value u and v are sine-like waves and can be selected as the output signal of the oscillator to control the joints. Only one signal is needed for every joint and u is chosen in this paper. To utilize this signal to control the rotation of the joint, the ascent stage of the signal is set corresponding to the stance phase, while the declining stage is set corresponding to the swing phase, as shown in Figure 6.

It could be found that in the configuration, as shown in Figure 5, the period of the stance phase will equal the period of the swing phase due to the symmetry of the output waveforms, which means this waveform is only suitable for tripod gait as its duty factor is 0.5. When in other gaits whose duty factor is not 0.5, a more complicated mapping relationship will be required to apply this signal to the robotic joint space.

**Figure 7:**
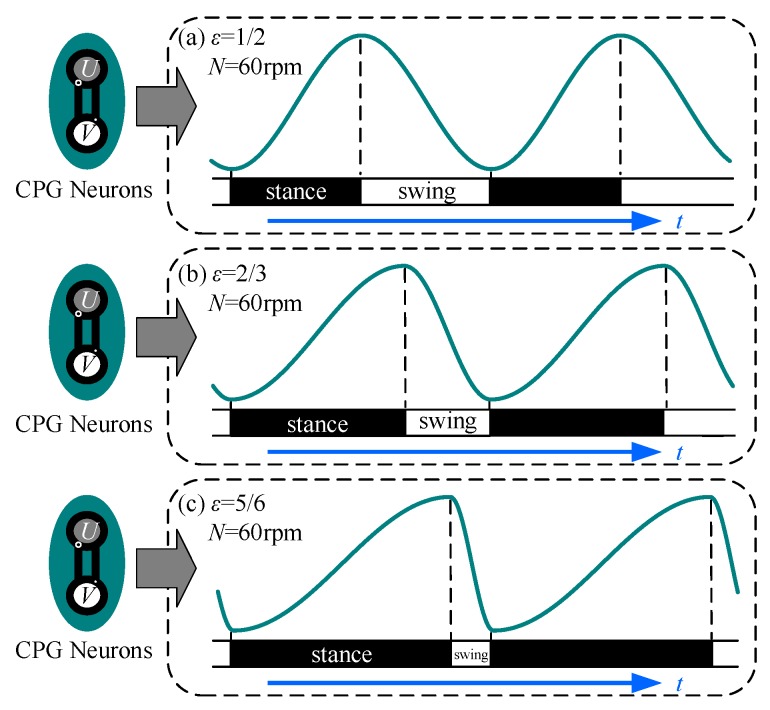
Correspondence between the modified oscillator and the locomotion phase: (**a**) *ε* = 1/2, *N* = 60 rpm; (**b**) *ε* = 1/2, *N* = 60 rpm; (**c**) *ε* = 5/6, *N* = 60 rpm. Other parameters: *σ* = 100, R = 1; The ratio of the ascent phase to the declining phase increases as the duty factor increases.

To solve this problem, a modified Hopf oscillator model is proposed in this paper. Because the phase difference of u and v is a quarter period, i.e., when u is in the ascent phase, v is a negative number; when u is in the declining phase, v will be a positive number. This relationship can be used to adjust the periods of the ascent phase and the declining phase, with the duty factor ε introduced into the model. In addition, the target averaged rotating speed of the joint can also be introduced to adjust the robotic moving speed. The modified model can be expressed by:

(14) $$\dot{X}=\left[\begin{array}{c} \dot{u}\\ \dot{v} \end{array}\right]=\left[\begin{array}{c} \sigma (R^{2}-u^{2}-v^{2})u-2\pi \omega_{k}v+f_{u}\\ \sigma (R^{2}-u^{2}-v^{2})v+2\pi \omega_{k}u+f_{v} \end{array}\right]$$

(15) $$\omega_{k}=\{\begin{array}{cc} \frac{N}{120(1-\varepsilon )} & (v_{k}\geq 0)\\ \frac{N}{120\varepsilon } & (v_{k}<0) \end{array}$$

where $v_{k}$ is the state value of v at time k; $\omega_{k}$ is the oscillating frequency at time k and it is adjusted by the duty factor; ε is the duty factor; N (rpm) is the averaged rotating speed. The correspondence between the signal generated by the modified model and the phase of the joint is shown in Figure 7. As shown in Figure 7, the ratio of the ascent phase to the declining phase increases as the duty factor increases.

The mapping relationship between the CPG output signal and the joint space can be expressed by:

(16) $$D_{k}(u_{k})=\left\{ \begin{array}{ll} \frac{\theta_{stance}}{2}\sin(\frac{\pi }{2}u_{k})+\frac{\theta_{stance}}{2} & \left(v_{k}\leq 0\right)\\ -\frac{\theta_{swing}}{2}\sin(\frac{\pi }{2}u_{k})+\frac{\theta_{swing}}{2}+\theta_{stance} & \left(v_{k}>0\right) \end{array}\right.$$

where *D_k_* represents the angle of the joint at time *k*.

**Figure 8:**
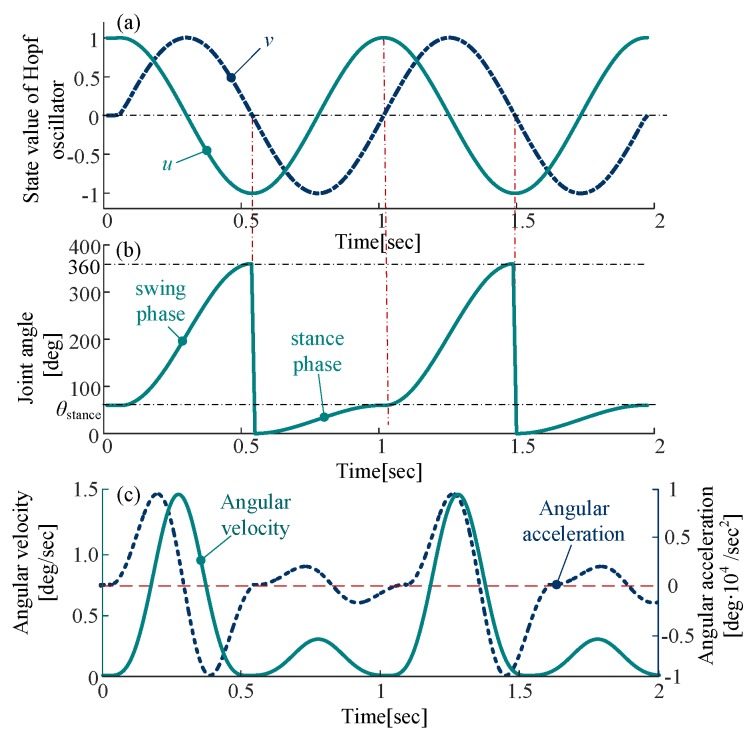
The mapping relationship between the output of Hopf oscillator and joint space: (**a**) state value of Hopf oscillator; (**b**) joint angle curve; (**c**) angular velocity and acceleration curve. The parameters of Hopf oscillator: *ε* = 1/2, *N* = 30 rpm, *σ* = 100, R = 1, *θ_stance_* = 30°.

This relationship is composed of trigonometric functions, the joint angle curve is smooth and periodic, as shown in Figure 8. The angular velocity curve and the angular acceleration curve obtained by derivation are also smooth without rigid impact and soft impact.

### 3.2. Gait Generation and Smooth Transition

**Figure 9:**
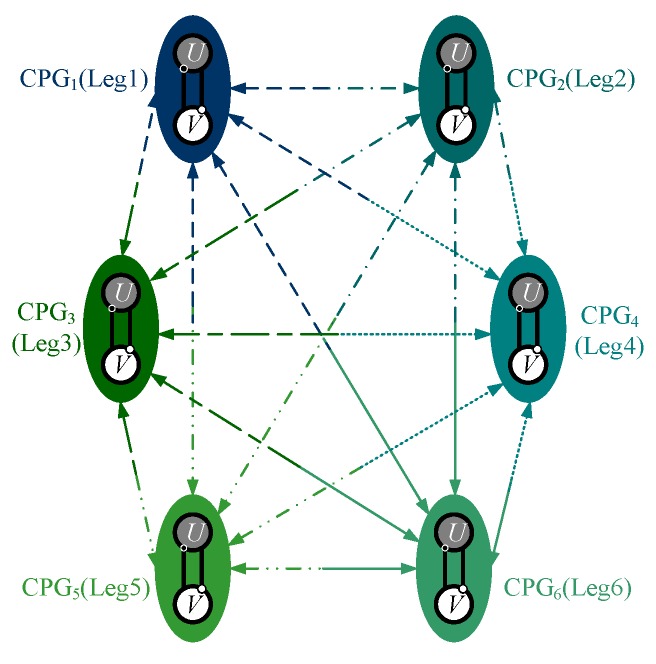
CPG network of six oscillators with a bidirectional coupling between every two oscillators.

**Figure 10:**
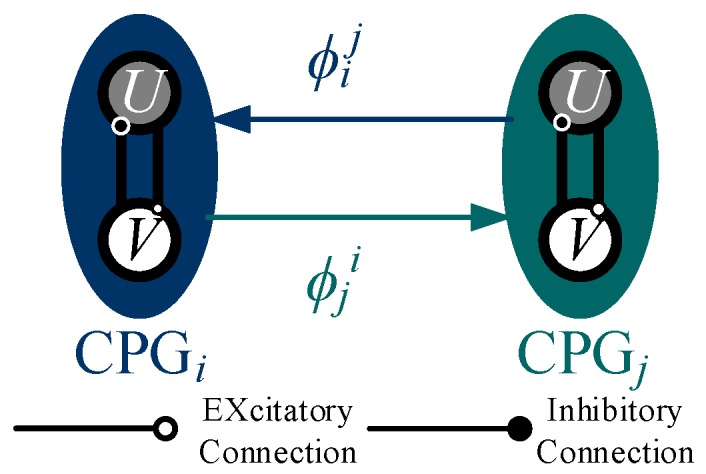
The bidirectional coupling between two oscillators.

Biological neurons can form a neural network via synaptic connections to control the behavior of living things. In this paper, the CPG control network is achieved by the nonlinear coupling of modified Hopf oscillators. Six joints are individually controlled by six oscillators in the CPG network. Because the total number of the joints is relatively small, the connection in the network model is chosen as the fully connected manner to guarantee phase stability and convergence speed, as shown in Figure 9, which means that there is a bidirectional coupling between every two oscillators, as shown in Figure 10.

As shown in Figure 10, $\phi_{i}^{j}$ represents the phase difference of the neuron oscillator j relative to the neuron oscillator i, the coupling term Δ between the two oscillators is expressed by:

(17) $$\Delta_{i}^{j}=\left[\begin{array}{cc} \cos\phi_{i}^{j} & -\sin\phi_{i}^{j}\\ \sin\phi_{i}^{j} & \cos\phi_{i}^{j} \end{array}\right]\left[\begin{array}{c} u_{j}\\ v_{j} \end{array}\right]$$

**Figure 11:**
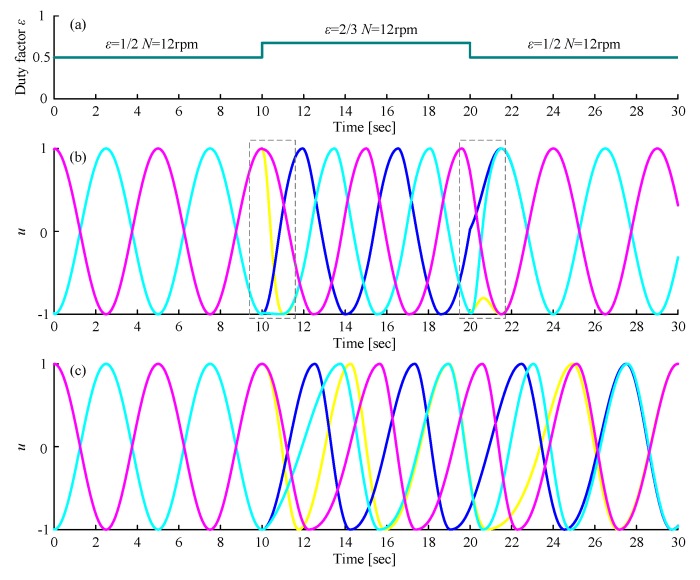
The comparison of the original gait transition method and the modified gait transition method: (**a**) Duty factor changes at time = 10 s and time = 20 s; (**b**) Output of the original method: distortion of the waveform appears after time = 10 s and time = 20 s; (**c**) output of the modified method: the distortion of the waveform after time = 10 s and time = 20 s disappears; Other parameters: *σ* = 100, R = 1.

In the CPG network, the oscillator coupled with other oscillators is added by the coupling term, i.e., the neuron oscillator i can be expressed by:

(18) $${\dot{X}}_{i}=\left[\begin{array}{c} {\dot{u}}_{i}\\ {\dot{v}}_{i} \end{array}\right]=\left[\begin{array}{c} \sigma (R^{2}-u_{i}{}^{2}-v_{i}{}^{2})u_{i}-2\pi \omega_{ik}v_{i}\\ \sigma (R^{2}-u_{i}{}^{2}-v_{i}{}^{2})v_{i}+2\pi \omega_{ik}u_{i} \end{array}\right]+\lambda \sum_{j}\Delta_{i}^{j}$$

(19) $$\omega_{ik}=\{\begin{array}{cc} \frac{N}{120(1-\varepsilon )} & (v_{ik}\geq 0)\\ \frac{N}{120\varepsilon } & (v_{ik}<0) \end{array}$$

where λ represents the coupling strength between oscillators. When the target phase difference, the averaged rotating speed of the joint and the duty factor are given, as referred to $\left(\phi_{1}^{2}\;\phi_{2}^{3}\;\phi_{3}^{4}\;\phi_{4}^{5}\;\phi_{5}^{6}\;\phi_{6}^{1}\;\varepsilon \;N\right)$, the outputs of six oscillators will automatically converge to the target signal waveform. Similarly, the gait transition is achieved by changing these parameters under the same network structure. However, this change may make the output waveform unsmooth and oscillate multiple times on one side of the X-axis, as shown in the dotted box in Figure 11b, which will produce an extremely disordered gait that will cause the robot to be in an unstable state.

A method is put forward to solve this problem: when the gait is going to be changed, the parameters can be changed continuously in a certain time from the current value to the target value via the following equations:

(20) $$\phi_{i}^{j}{}^{+}=\phi_{i}^{j}{}^{+}+\frac{\phi_{i}^{j}{}^{-}-\phi_{i}^{j}{}^{+}}{e^{\kappa (t-t_{0})}}$$

(21) $$\varepsilon^{+}=\varepsilon^{+}+\frac{\varepsilon^{-}-\varepsilon^{+}}{e^{\kappa (t-t_{0})}}$$

where $\phi_{i}^{j}{}^{+}$ and $\varepsilon^{+}$ are the target value; $\phi_{i}^{j}{}^{-}$ and $\varepsilon^{-}$ are the current value; $t_{0}$ represents the period getting the instruction to change; t is the current time from $t_{0}$; κ can be utilized to adjust the transition time. When it is to change from a gait with a small duty factor to a gait with a large duty factor, for example, change from the tripod gait to the quadruped gait, κ should be set small; otherwise, κ should be set large. Figure 11c shows the transition stage of our proposed method, which avoided the above problem.

### 3.3. Gait planning Based on CPG Control Method

**Figure 12:**
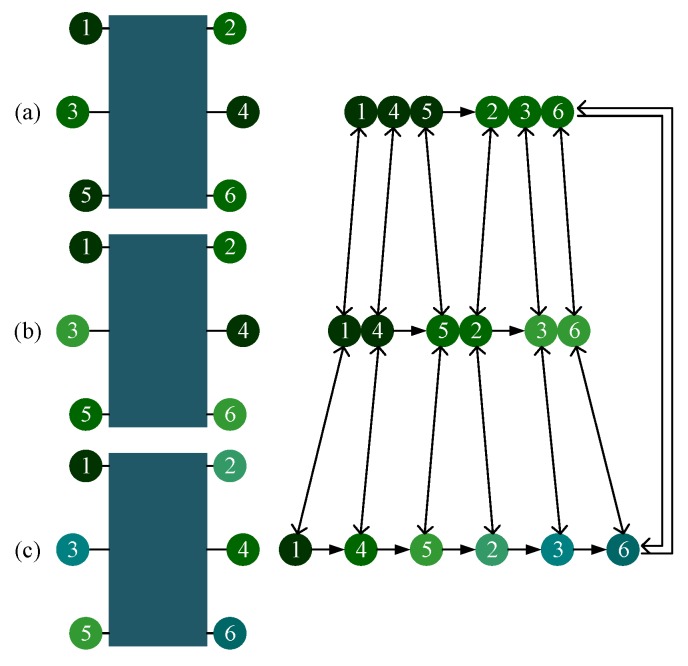
Gait planning based on CPG control: (**a**) Tripod gait: Leg 1, Leg 4 and Leg 5 form the left supporting triangle, Leg 2, Leg 3 and Leg6 form the right triangle, these two groups of legs support and swing alternately; (**b**) Quadruped gait: Leg1 and Leg 4 form the first group, Leg 5 and Leg 2 form the second group, Leg 3 and Leg6 form the third group, there three groups of legs support and swing in the order shown by the arrow; (**c**) Wave gait: all the six legs support and swing in the order shown by the arrow.

CPG network can generate different signals for different gaits by adopting different parameters. To avoid the unstable situations analysed above, the robotic gait ought to be planned reasonably. When in tripod gait, the six legs are divided into two groups: the left support triangle (Leg 1, Leg 4, and Leg 5) and right support triangle (Leg 2, Leg 3 and Leg 6), as shown in Figure 12a. These two groups of legs will stay in the stance and swing phases alternately to achieve the locomotion of the robot. There will always be three legs in the stance phase while the other three legs in the swing phase at any time. The phase difference between legs of different groups is half of 2π, and the duty factor is 0.5, so the value of gait parameters $\left(\phi_{1}^{2}\;\phi_{2}^{3}\;\phi_{3}^{4}\;\phi_{4}^{5}\;\phi_{5}^{6}\;\phi_{6}^{1}\;\varepsilon \right)$ are set as (−π 0 π 0 −π π 0.5).

When in quadruped gait, the six legs are divided into three groups to swing, with four legs in the stance phase at any time. Considering the converging process from tripod gait to quadruped gait, to minimize the change, Leg 5 from the left support triangle and Leg 2 from the right support triangle in the tripod gait will form a new group, and so on. In this way, the relative positional relationship of six legs in the sequence has not changed. It can not only reduce the time used to converge into the target gait, but also ensure that the above instability state does not occur during the transition.

**Figure 13:**
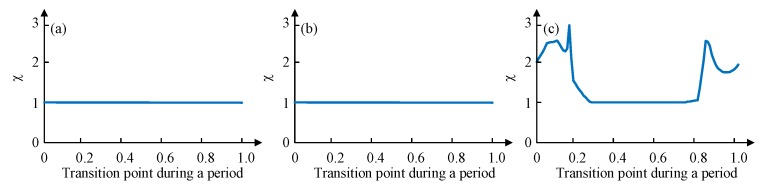
Transitions disorder tests from MATLAB simulations: (**a**) Tripod-quadruped transition using the proposed method; (**b**) Quadruped-tripod transition using the proposed method; (**c**) Tripod-quadruped transition using the conventional method.

The phase difference between legs of different group is 1/3 of 2π and the duty factor is 2/3, according to the leg sequence planned, the values of gait parameters $\left(\phi_{1}^{2}\;\phi_{2}^{3}\;\phi_{3}^{4}\;\phi_{4}^{5}\;\phi_{5}^{6}\;\phi_{6}^{1}\;\varepsilon \right)$ are set as (−π −π/3 π −π/3 −π −π/3 0.67). A MATLAB simulation of switching from tripod gait to quadruped gait at any time during the whole period of tripod (0 to 1, the accuracy of 0.01) is tested. Once an unstable state mentioned previously happens during the transition, χ will increase 0.01. Figure 13 depicts the simulation results.

Figure 13a shows the disorder degree of switching from tripod gait to quadruped gait using the proposed gait planning method, and Figure 13b shows the disorder degree of switching from quadruped gait to tripod gait using the proposed gait planning method. Both of them stay unchanged all the time, which proves the effectiveness of our proposed gait planning. Figure 13c shows the disorder degree of switching from tripod gait to quadruped gait using the other quadruped gait planning method mentioned. The result suggests that there will be an unstable situation happening during the transition if the switch at some points.

**Figure 14:**
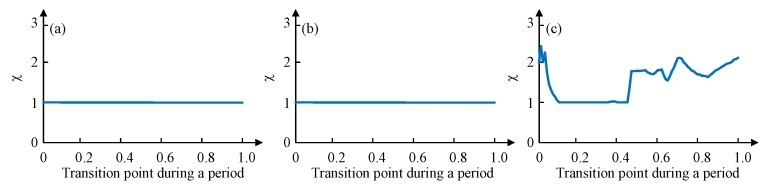
Transitions disorder tests from MATLAB simulations: (**a**) Quadruped-wave transition using the proposed method; (**b**) Wave-quadruped transition using the proposed method; (**c**) Quadruped-wave transition using the conventional method.

When in the wave gait, there will be five legs in the stance phase at any time. To plan the wave gait, and to achieve the least change during the mutual transition of quadruped and wave gait, two legs of each group of the quadruped gait are separated, and the relative positional relationship of six legs in the sequence remains unchanged. The phase difference between every two sequentially connected legs is 1/6 of 2π and the duty factor is 5/6, according to the leg sequence planned, the values of gait parameters $\left(\phi_{1}^{2}\;\phi_{2}^{3}\;\phi_{3}^{4}\;\phi_{4}^{5}\;\phi_{5}^{6}\;\phi_{6}^{1}\;\varepsilon \right)$ are set as (−2π/3 −2π/3 4π/3 −2π/3 −2π/3 4π/3 0.83). This gait planning method reduces the disorder degree of the phase transition and can avoid the unstable situation, as shown in Figure 14a,b. Another wave gait planning method is compared: swing from Leg 1 to Leg 6 in order, and the result is shown in Figure 14c.

**Figure 15:**
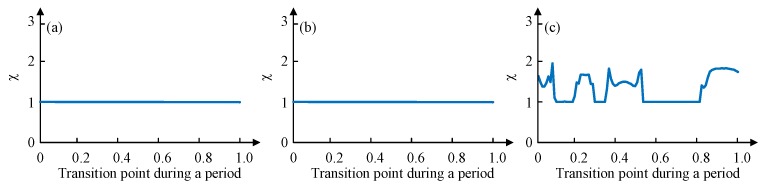
Transitions disorder tests from MATLAB simulations: (**a**) Tripod-wave transition using the proposed method; (**b**) Wave-tripod transition using the proposed method; (**c**) Tripod-wave transition using the conventional method.

Similarly, the mutual transition between the tripod and the wave gait using the proposed gait planning is tested and compared. As shown in Figure 15, the results also certify the stability of the proposed method in gait transition.

## 4. Locomotion Simulation and Experiment

**Figure 16:**
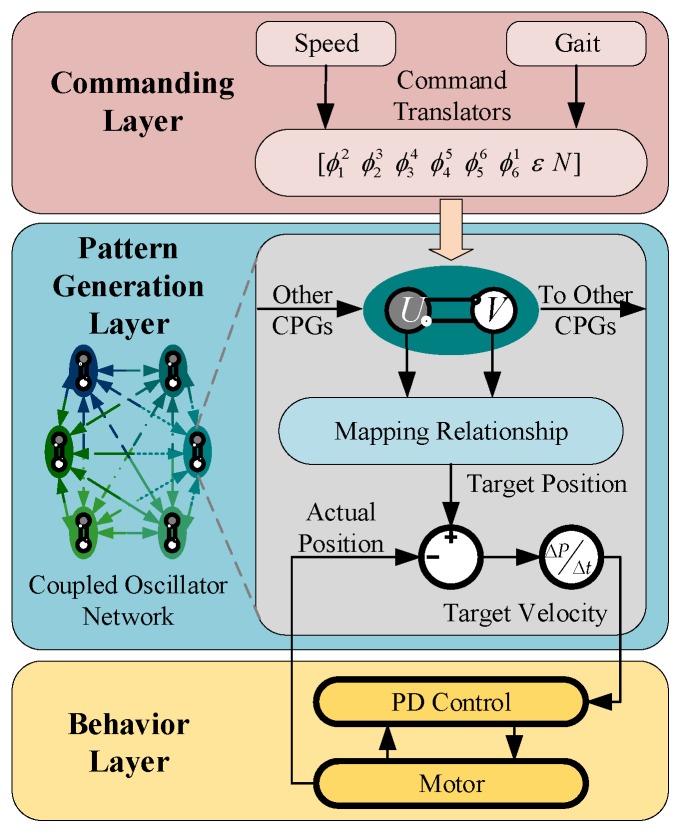
The locomotion control system based on CPG. Instructions from the commanding layer are translated into parameters to the pattern generation layer. Then the control signal is produced by the CPG network and mapped to the robotic joint space.

For further verification, a locomotion simulation based on Simulink and ADAMS and prototype experiments are carried out. The locomotion control system based on CPG is shown in Figure 16. Instructions from the commanding layer are translated into parameters to the pattern generation layer. Then the control signal is produced by the CPG network and mapped to the robotic joint space.

### 4.1. Mutual Transitions of Tripod and Quadruped Gait

**Figure 17:**
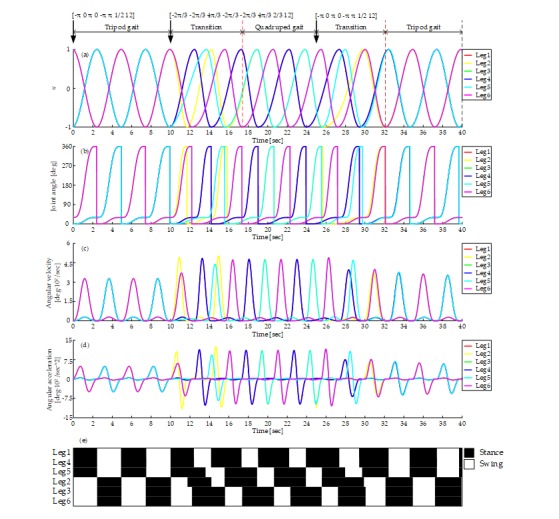
Gait generation of the tripod, the quadruped gait, and the mutual transitions: (**a**) Output of CPG network; (**b**) Joint angle curve; (**c**) Angular velocity curve; (**d**) Angular acceleration curve; (**e**) Gait diagram, represents the CPG output signals, black and white areas denote the stance and swing phase respectively.

Firstly, the simulation and experiment of mutual transition of tripod and quadruped gait are carried out. Figure 17a shows the output of the CPG network. It starts with tripod gait and switches to quadruped at time = 10 s. About 7.5 seconds are used to converge into the quadruped gait. And then the transition from quadruped gait to tripod gait happened at time = 25 s. About 7 seconds are used to converge into the tripod gait. The average input rotational speed is 12 rpm all the time. During the whole process, the curve is smooth without any disorders mentioned above.

As shown in Figure 17b–d, the joint angle curve, angular velocity curve and angular acceleration curve are all smooth, which proves that there is no rigid and soft impact on motors during the process of tripod gait, quadruped gait and their mutual transition using the proposed mapping relationship. The angular velocity and acceleration of the swing phase are higher than that of the stance phase for that swing angle is bigger than the stance angle and time of swing phase is less. The peak ratio of rotate velocity equals to the product of duty factor and the ratio of swing and stance angle. However, the load in the swing phase is much less than that in the stance phase. Figure 17e shows the gait diagram, it is consistent with the gait planned when in the stages of the tripod gait and the quadruped gait, and during the transitions, there are enough legs supporting the body and no unstable states at any time.

**Figure 18:**
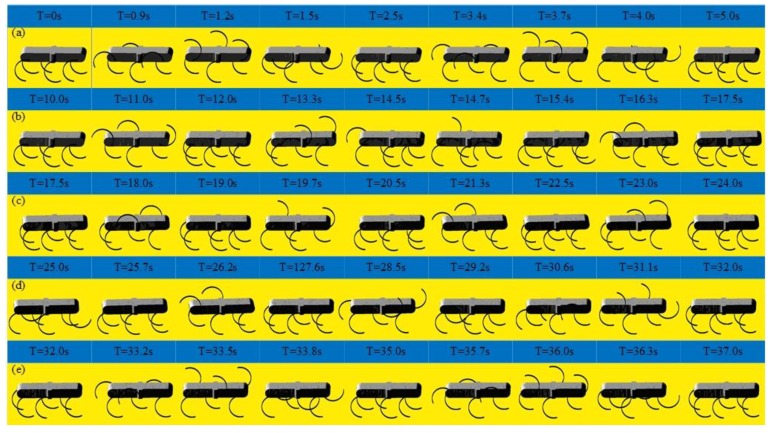
Locomotion simulation of the tripod, the quadruped gait, and the mutual transitions: (**a**) A period of tripod gait; (**b**) Tripod-quadruped transition; (**c**) A period of quadruped transition; (**d**) Quadruped-tripod transition; (**e**) A period of tripod gait after transitions.

As shown in Figure 18, the locomotion simulation of the tripod gait, quadruped gait and the smooth and stable transition between them are achieved successfully. As shown in Figure 18a, when the legs of the left support triangle are in stance phase, the legs of the right support triangle are in swing phase, when the legs of the left support triangle are in swing phase, the legs of the right support triangle are in the stance phase. At any time, there are three legs in stance phase. When in the tripod-quadruped transition, as shown in Figure 18b, the rotational speed of Leg 2 is improved compared with the other legs of the right support triangle, the rotational speed of Leg 5 is reduced compared with the other legs of the left support triangle. Then Leg 2 and Leg 5 are synchronized, and quadruped gait is formed, as shown in Figure 18c, three groups of legs (Leg 1 and Leg 4, Leg 2 and Leg 5, Leg 3 and Leg 6) swing in turn, and at any time, there are four legs in stance phase. When in quadruped-tripod transition, as shown in Figure 18d, Leg 2 slows down to keep pace with Leg 3 and Leg 6 to form right support triangle, Leg 5 speeds up to keep pace with Leg 1 and Leg 4 to form left support triangle. Then tripod gait is achieved, as shown in Figure 18e. During this simulation, the robotic average velocity is 0.048 m/s in the tripod gait and 0.036 m/s in the quadruped gait.

**Figure 19:**
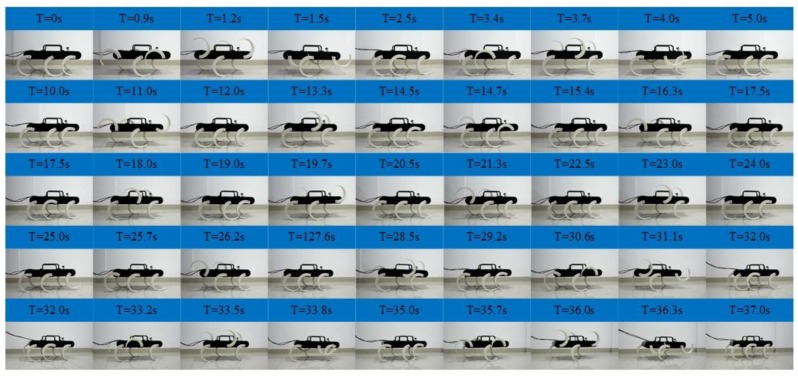
Locomotion experiment of tripod and quadruped gait and the mutual transitions.

As shown in Figure 19, experiments of the tripod, quadruped gait and mutual transitions were implemented. It is in the tripod gait in the first 10 s and then switches to the quadruped gait by online adjusting the parameters of the CPG network. The quadruped gait is realized at time =17.5 s and continues to time = 25 s and then switches back to the tripod gait via online adjusting the parameters. It converges back into tripod gait at time = 32 s and continues to time = 40 s. The tripod-quadruped and quadruped-tripod transitions are achieved by online adjusting the parameters of the CPG network, and the transitions are stable and continuous without pause. The locomotion in the experiment is in agreement with the result of the simulation and the output signal of the CPG network. The effectiveness of the proposed method in the transitions between the tripod and the quadruped gait is further verified. The average velocity is about 0.04 m/s in the tripod gait and about 0.03 m/s in the quadruped gait.

### 4.2. Mutual Transitions of Tripod and Wave Gait

**Figure 20:**
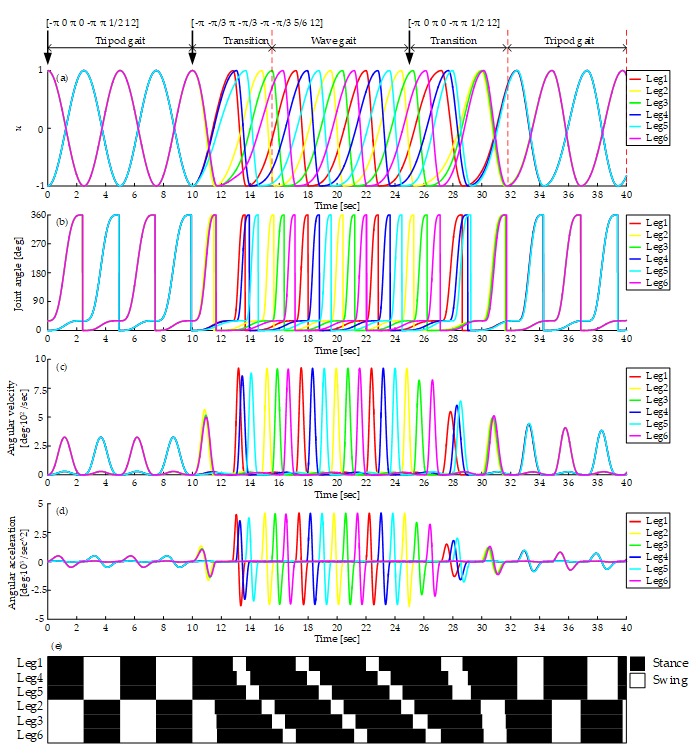
Gait generation of the tripod, the wave gait, and the mutual transitions: (**a**) Output of CPG network; (**b**) Joint angle curve; (**c**) Angular velocity curve; (**d**) Angular acceleration curve; (**e**) Gait diagram, represents the CPG output signals, black and white areas denote the stance and swing phase respectively.

Figure 20a shows the output of the CPG network in the tripod, the wave gait, and mutual transitions. It starts with the tripod gait and switches to the wave gait at time = 10 s. About 5.5 seconds are used to converge into wave gait. Then the transition from the wave gait to the tripod gait happens at time = 25 s. About 6.8 seconds are needed to converge to the tripod gait. The average input rotational speed is 12 rpm all the time. The curve of the output signal of the CPG network is smooth during the whole process.

Figure 20b–d shows the curves of joint angle, angular velocity, and angular acceleration. The smooth curves show that there is no rigid and soft impact on motors using this method. The peak angular velocity and acceleration of wave gait are higher for its bigger duty factor. Figure 20e shows the gait diagram, it is consistent with the gait planned when in the stages of the tripod gait and the wave gait, and during the transitions, there are enough legs supporting the body and no unstable states at any time.

**Figure 21:**
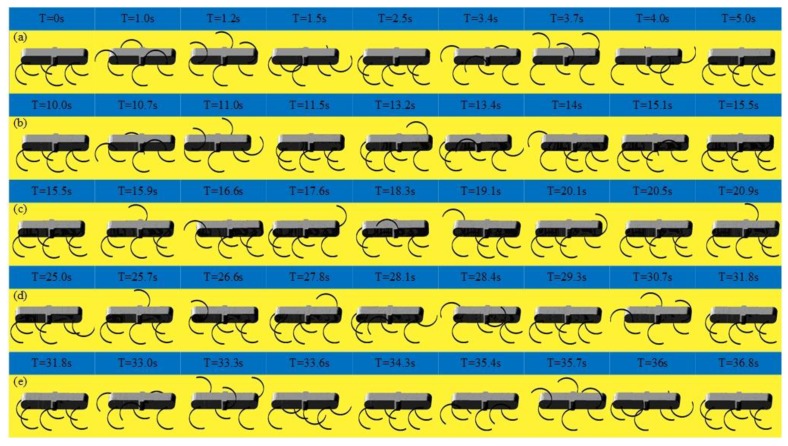
Locomotion simulation of the tripod, the wave gait, and the mutual transitions: (**a**) A period of tripod gait; (**b**) Tripod-wave transition; (**c**) A period of wave transition; (**d**) Wave-tripod transition; (**e**) A period of tripod gait after transitions.

As shown in Figure 21, the locomotion simulation of the tripod, the wave gait, and the mutual transitions is carried out. It starts with the tripod gait, as shown in Figure 21a. When in the tripod-wave transition, the angular velocity of legs of the same support triangle become different and the phase difference increases, as shown in Figure 21b. And then six legs swing in turn. At any time, there are five legs in the stance phase, as shown in Figure 21c. When in wave-tripod transition, the phase difference between legs of the same support triangle reduces to 0 gradually, as shown in Figure 21d. Then the tripod gait is achieved, as shown in Figure 21e. The average velocity during this simulation is 0.048 m/s in the tripod gait and 0.029 m/s in the wave gait.

**Figure 22:**
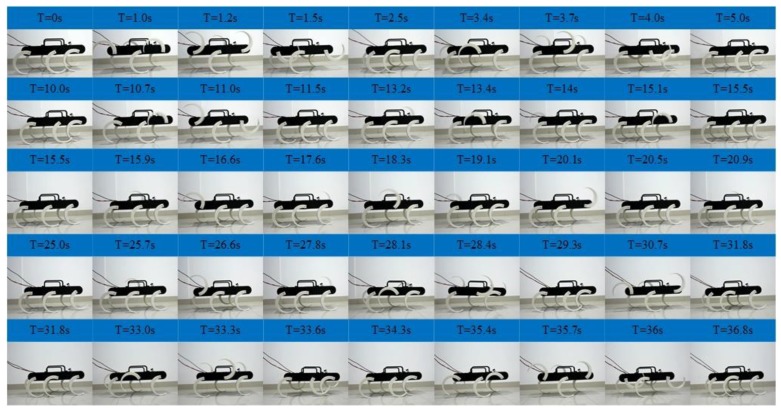
Locomotion experiment of the tripod, the wave gait, and the mutual transitions.

Figure 22 shows the result of the experiment of the tripod, the wave gait, and the mutual transitions. It is in the tripod gait in the first 10 s and then switches to the wave gait by online adjusting the parameters of the CPG network. The wave gait is achieved at time = 15.5 s and continues to time = 25 s and then switches back to the tripod gait by online adjusting the parameters. It converges back into the tripod gait at time = 31.8 s and continues to time = 40 s.

The process agrees with the simulation results and the output signal of the CPG network. The transitions are achieved smoothly and stably, and there is no unstable situation and pause happening. The effectiveness of the proposed method in the transitions between the tripod and the wave gait is further verified. The average velocity during this experiment is about 0.04 m/s in the tripod gait and 0.02 m/s in the wave gait.

### 4.3. Mutual Transitions of Quadruped and Wave Gait

**Figure 23:**
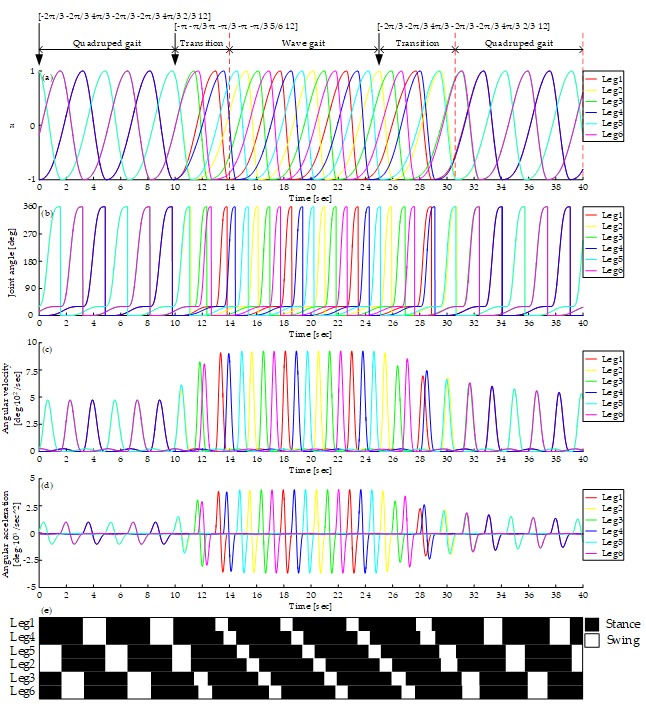
Gait generation of the quadruped, the wave gait, and the mutual transitions: (**a**) Output of CPG network; (**b**) Joint angle curve; (**c**) Angular velocity curve; (**d**) Angular acceleration curve; (**e**) Gait diagram, represents the CPG output signals, black and white areas denote the stance and swing phase, respectively.

Figure 23a shows the output of the CPG network in the quadruped, the wave gait, and mutual transitions. The simulation starts with the quadruped gait and switches to the wave gait at time =10 s, the transition uses 4s to converge into the wave gait and then switches from the wave gait to the quadruped gait at time = 25 s, where the transition uses 5.6 s. The average input rotational speed is 12 rpm all the time. The curve of the output signal of the CPG network is smooth during the whole process.

Figure 23b–d show the curves of joint angle, angular velocity, and angular acceleration. The smooth curves show that there is no rigid and soft impact on motors using this method. Figure 23e shows the gait diagram, it is consistent with the gait planned when in the stages of the quadruped gait and the wave gait, and during the transitions, there are enough legs supporting the body and no unstable states at any time.

**Figure 24:**
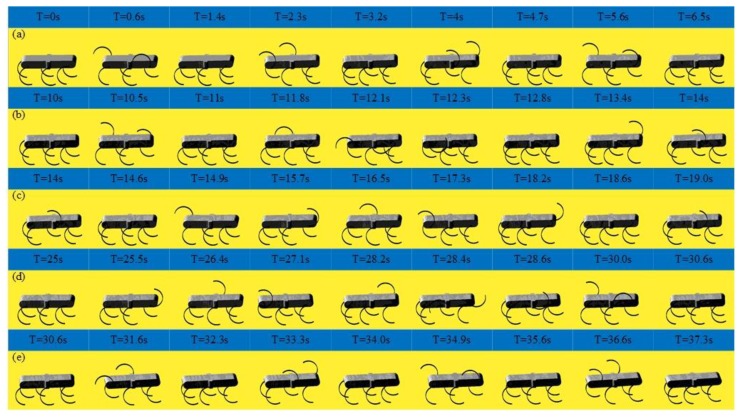
Locomotion simulation of the quadruped, the wave gait, and the mutual transitions: (**a**) A period of quadruped gait; (**b**) Quadruped-wave transition; (**c**) A period of wave transition; (**d**) Wave-quadruped transition; (**e**) A period of quadruped gait after transitions.

As shown in Figure 24, the locomotion simulation of the quadruped, the wave gait, and the mutual transitions is carried out. It starts with the quadruped gait, as shown in Figure 24a. When in the quadruped-wave transition, the angular velocity of legs of each group become different, and the phase difference increases from 0, as shown in Figure 24b. Then six legs swing in turn. At any time, there are five legs in the stance phase, as shown in Figure 24c. When in wave-tripod transition, the phase difference between the legs of each group reduces to 0 gradually, as shown in Figure 24d. Then the quadruped gait is achieved, as shown in Figure 24e. The average velocity during this simulation is 0.036 m/s in the quadruped gait and 0.029 m/s in the wave gait.

**Figure 25:**
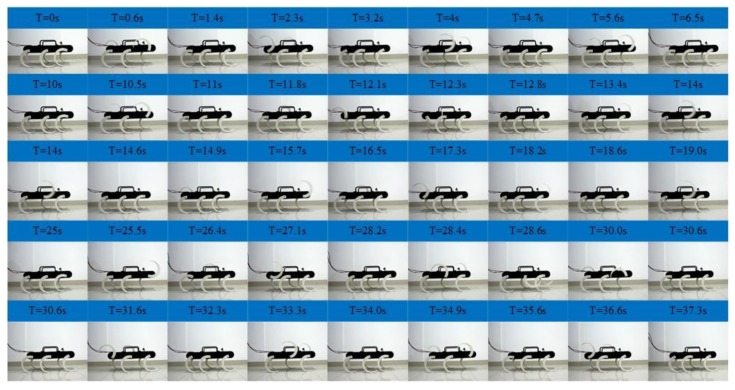
Locomotion experiment of the quadruped, the wave gait, and the mutual transitions.

Figure 25 shows the result of the experiment of the quadruped, the wave gait, and the mutual transitions. It is in the quadruped gait in the first 10 s and then switches to the wave gait by adjusting online the parameters of the CPG network. The wave gait is achieved at time = 14 s and continues to time = 25 s and then switches back to the quadruped gait by adjusting online the parameters. It converges back into the quadruped gait at time = 30.6 s and continues to time = 40 s. The process agrees with the result of simulation and the output signal of the CPG network. The transitions are achieved smoothly and stably, and no unstable situations and pauses happen. The effectiveness of the proposed method in the transitions between the quadruped and the wave gait is further verified. The average velocity during this experiment is 0.03m/s in the quadruped gait and 0.02 m/s in the wave gait.

### 4.4. Transitions on Uneven Ground

**Figure 26:**
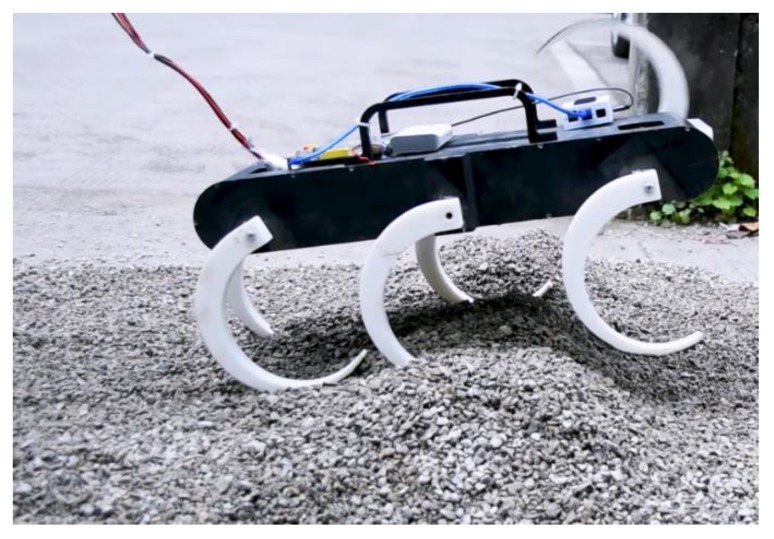
Uneven ground paved with rubble.

**Figure 27:**
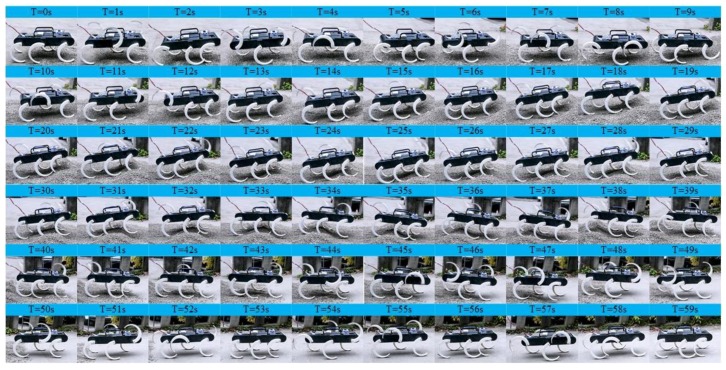
Locomotion and gait transition on uneven ground.

Figure 26 shows the uneven ground paved with the rubble and Figure 27 shows the experimental results of walking and transitions on uneven pavement. In the beginning, the robot walks on the gravelly ground in the tripod gait. Then a pile of rubble appears in front of the robot, so the robot switches into the wave gait at time = 10 s to pass through this rugged terrain. At this stage, the body is tilted, and some supporting legs are not on the ground due to the large height difference of the terrain. After passing through the obstacles, the robot switches into the quadruped gait at time = 41 s to go through the transition area between the gravelly ground and flat ground. Finally, the robot switches back into the tripod gait at time = 51 s to walk on a slope, and this stage lasts until time = 60 s. In the whole process, the gait transitions are fast and smooth, and the robot walks stably, which suggests that the gait transition proposed in this paper can be used to adapt to the different ground for the robot in the uneven entertainment.

### 4.5. Velocity Transition in the Tripod Gait

**Figure 28:**
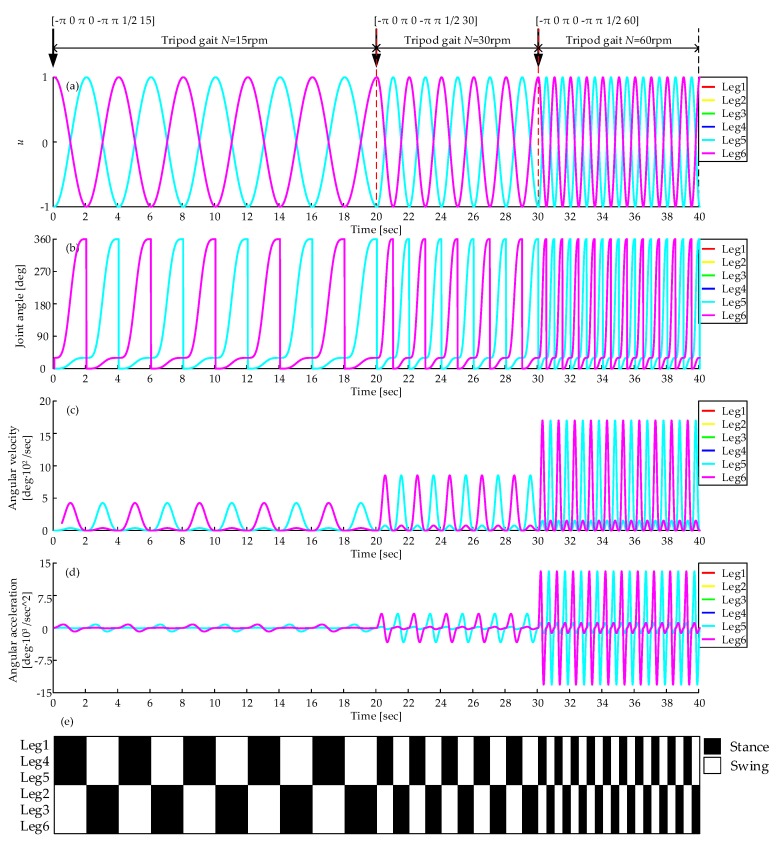
Velocity transition in the tripod gait: (**a**) Output of CPG network; (**b**) Joint angle curve; (**c**) Angular velocity curve; (**d**) Angular acceleration curve; (**e**) Gait diagram, represents the CPG output signals, black and white areas denote the stance and swing phase respectively.

The robotic velocity can be switched by adjusting the average input rotational speed *N* (rpm). As shown by Equation (15), different rotational speeds can produce different oscillation frequencies, corresponding to different velocity, and the relationship is expressed by Equation (12). Figure 28a shows the CPG output with different parameter *N* (rpm). The whole process is in the tripod gait and starts with *N* = 15 rpm, and switches *N* to 30 rpm and 60 rpm at time = 20 s and time = 30s respectively. As *N* increases, the frequency of the output waveform increases. Figure 28b–d show the curves of joint angle, angular velocity, and angular acceleration. The period of the curves of joint angle reduces and the peak of the angular velocity and angular acceleration increase. Figure 28e shows the gait diagram, the velocity transition is quick and smooth, and with the average input rotational speed increasing, the cycle time is decreasing.

**Figure 29:**
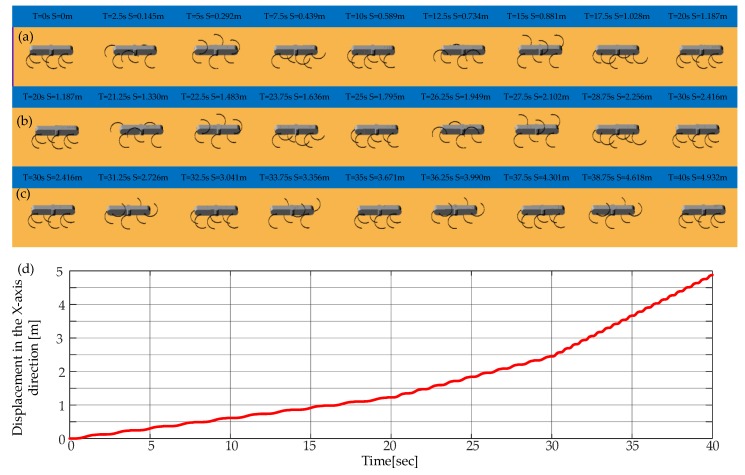
Locomotion simulation of the velocity transition in the tripod gait: (**a**) *N* = 15 rpm,; (**b**) *N* = 30 rpm; (**c**) *N* = 60 rpm; (**d**) the displacement during the simulation.

As shown in Figure 29, the locomotion simulation of the velocity transition in the tripod gait is carried out. Figure 29a–c show three stages, respectively. Figure 29d shows the displacement in the forward direction, and the average velocities of these three stages are 0.06 m/s, 0.12 m/s and 0.25 m/s, which is consistent with Equation (12).

**Figure 30:**
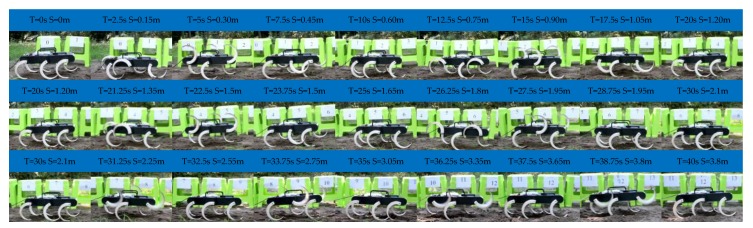
Locomotion experiment of the velocity transition in the tripod gait.

Figure 30 shows the result of the experiment of the velocity transition in the tripod gait. The distance between each two markers is 0.3 meters, and the displacement of robot is recorded with the precision of half of the distance between each two makers. The gait in the experiment is consistent with the simulation, and the displacement is recorded in the figures. The average velocities of three stages in this experiment are about 0.06 m/s, 0.09 m/s and 0.17 m/s which are less than in the simulation. The possible reason is that the phenomenon of slippage during the experiment is more serious.

## 5. Conclusions

In this paper, the design, control, and experiments of the curved-leg hexapod robot are presented. Firstly, the locomotion and support stability of the curved-leg robot are analyzed, from which analysis results the parameters of the curved leg is obtained. Then, the original Hopf oscillator is modified by introducing a duty factor to generate asymmetric waveforms that are suitable for various gaits, and a variable CPG network model that can achieve seamless gait transition is built for gait control. To smooth the transition of gait switching, new gait transition equations and gait planning are proposed, the parameters of which can change from the current value to the target value continuously and smoothly. Finally, the tripod gait, the quadruped gait, and the wave gait, as well as all the mutual gait transitions, are tested in Adams simulations and practical experiments. The results show that the proposed method can realize smooth gait transitions between each gait continuously, which improves the applicability of CPG in the gait control of the hexapod robot. Further work will focus on the introduction of force feedback and the dynamic model to the CPG model to improve the adaptability in all kinds of terrain while maintaining the smooth and stable gait transition.

## Figures and Tables

**Table 1:** Length of the structure parameters.

Name	*W*_n_ (mm)	*W*_b_ (mm)	*D* (mm)	*R* (mm)	*Ρ* (mm)
**Value**	280	320	230	110	210

**Table 2:** Distances from the centroid projection to each edge of the supporting polygons in the tripod gait.

Distance	$d_{14}$	$d_{15}$	$d_{23}$	$d_{26}$	$d_{36}$	$d_{45}$
**Value (mm)**	93	140	93	140	93	93

**Table 3:** Distances from the centroid projection to each edge of the supporting polygons in the quadruped gait and the wave gait.

Distance	$d_{12}$	$d_{13}$	$d_{14}$	$d_{15}$	$d_{23}$	$d_{24}$	$d_{26}$	$d_{35}$	$d_{36}$	$d_{45}$	$d_{46}$	$d_{56}$
**Value (mm)**	230	159	93	140	93	159	140	159	93	93	159	230
